# Light and nutrient limitations for tree growth on young versus old soils in a Bornean tropical montane forest

**DOI:** 10.1007/s10265-020-01217-9

**Published:** 2020-08-07

**Authors:** Shin-ichiro Aiba, Kanehiro Kitayama

**Affiliations:** 1 Faculty of Environmental Earth Science, Hokkaido University, Sapporo, 060-0810 Japan; 2 Graduate School of Agriculture, Kyoto University, Kyoto, 606-8502 Japan

**Keywords:** Beta diversity, Floristic turnover, Pedogenesis, Soil aging, Tree growth

## Abstract

We examined forest and tree responses to decreasing nutrient availability with soil aging in a species-rich tropical montane rain forest on Mount Kinabalu, Borneo. Community composition and structure and tree growth rates were compared between two 1 ha plots on nutrient-rich young soil versus nutrient-deficient old soil. Myrtaceae and Fagaceae dominated both plots. With soil aging, the dominance of Lauraceae, stem density, basal area and aboveground biomass decreased, and the forest understory became brighter. Some dominant taxa on the old soil (Podocarpaceae and the genus *Tristaniopsis* in Myrtaceae) were virtually absent on the young soil; this was attributed to light limitation in the understory. Growth rates of understory trees were lower on the young soil, whereas those of canopy trees were lower on the old soil. This suggested that the growth of understory trees was limited by light on the young soil, whereas that of canopy trees was limited by nutrients on the old soil. Of the eight species that were abundant in both plots, the dominance of five species was considerably lower on the old soil, four of which also exhibited decreased maximum sizes and lower growth rates. The remaining three species showed similar dominance across plots without a decline in growth rates, although they exhibited decreased maximum sizes on the old soil. These analyses demonstrated divergent responses of species to the soil-age gradient. We suggest that the differential responses of species to decreasing nutrient availability with a concomitant increase in understory light levels explain floristic turnover with soil aging.

## Introduction

Tropical rain forests are home to a high diversity of plants, which are associated with a rapid rate of spatial species turnover or ‘beta diversity’ (Condit et al. 2002; Slik et al. 2009). Soil nutrient conditions are an important cause of beta diversity among tropical rain forest stands under the same climatic conditions (John et al. 2007; Potts et al. 2002). Soil properties are determined by five soil-forming factors, i.e. climate, organisms, topography, parent material and time (Jenny 1941). When the first four factors are controlled, the soil properties change as a function of time since the onset of soil formation. Under such conditions, the availability of soil nutrients that are mostly derived from the parent material, such as phosphorus, will change according to the model proposed by Walker and Syers (1976). Nutrient concentrations are greatest in young soil but decline continuously during pedogenesis as nutrients are lost by leaching (and are converted to forms unavailable to plants, as in the case of phosphorus) at a greater rate than they are replenished by the parent material (Turner and Condron 2013). Many studies have analyzed the spatial variation of tropical forests along soil gradients related to topography (John et al. 2007; Takyu et al. 2002) and parent materials (Aiba and Kitayama 1999; Potts et al. 2002). However, few studies have examined the variation of tropical forests along a soil-age gradient.

Stunted vegetation occurs on severely nutrient-impoverished soil at the late stage of pedogenesis (Kitayama et al. 1997; Wardle et al. 2004). Competition for light in such vegetation on infertile soil will be less severe than in dense vegetation on fertile soil (Laliberté et al. 2013) because the plant canopy is predicted to have a greater leaf area under higher nutrient availability (Hikosaka 2003). Therefore, the limiting factor for juvenile tree growth in the forest understory shifts from light to nutrients along a gradient of decreasing soil nutrients (Andersen et al. 2014). Similarly, the growth of canopy trees receiving full sunlight is increasingly limited by the lack of nutrients with soil aging, whereas they are generally not limited by light irrespective of soil age. Thus, light limitation in the forest understory is more severe on young soils, whereas nutrient limitation for canopy trees is more severe on old soils. This could explain species turnover through pedogenesis, given that a trade-off exists between the use efficiency of light versus that of nutrients (the efficiency of carbon gain per unit light or nutrients that plants gain). There are no ‘superspecies’ (Tilman 1982) that are highly efficient in the use of both light and nutrients. Many studies suggest the physiological mechanisms underlying the trade-off between the use efficiency of light versus soil nutrients (e.g. Baltzer and Thomas 2007; Dent and Burslem 2016; Givnish 1988; Russo et al. 2008). Increased nitrogen supply will increase the light use efficiency of both sun leaves (by increasing allocation to Rubisco per unit leaf area, Evans 1989) and shade leaves (by increasing allocation to chlorophyll, Niinemets 1997). In contrast, species adapted to nutrient-deficient soil could not have high light use efficiency because of increased energy and nutrient investment to roots or towards defense against herbivores (Fine et al. 2006; Givnish 1988).

Based on the above considerations, we hypothesize the following scenario to explain floristic turnover with soil aging. On young soil, aboveground tree growth is enhanced and canopy cover increases. This reduces light levels in the forest understory, and the growth of juvenile trees becomes more limited by light than by soil nutrients. Environmental filtering will select shade-tolerant species with high light use efficiency that can maintain a positive carbon budget under deep shade. Such species should be nutrient-demanding because of the above-mentioned trade-off. With increasing soil age, soil nutrients decrease, which leads to a decline in growth rates and canopy cover. This increases light levels in the understory, and tree growth becomes more limited by nutrients than by light. Environmental filtering will select light-demanding species that can grow on nutrient-impoverished soil. Thus, it is predicted that understory trees grow more slowly and shade-tolerant species dominate on young soil because of light limitation, whereas canopy trees grow more slowly and light-demanding species dominate on old soil because of nutrient limitation. A comparative study across sites at different stages of soil aging (a so-called chronosequence study, Walker et al. 2010) is useful to test this scenario, which could account for the observed beta diversity.

In this paper, we ask how the forest and trees respond to decreasing nutrient availability during pedogenesis in a species-rich tropical forest. For this, forest composition and structure of the two 1-ha plots on soils of greatly different ages are described to demonstrate floristic turnover and associated change in physiognomy. Then tree growth is analyzed at the stand and species levels in relation to light and nutrient limitations. Finally, the implications of the results are discussed to test the above-mentioned scenario of the floristic turnover with soil aging, which could generate the high beta diversity observed in tropical forests.

## Materials and methods

### Study sites

Mount Kinabalu (6° 05′ N, 116° 33′ E), Malaysian Borneo, the highest mountain (4095 m asl) in South-East Asia, is one of the global hotspots of plant diversity (Beaman 2005). It is a non-volcanic mountain with diverse geology. Geological diversity is greatest in the lower montane zone (Aiba et al. 2015), which has three types of parent material, i.e. ultrabasic rock of Jurassic–Cretaceous age, sedimentary rock of Tertiary origin (Eocene–Early Miocene, 48–18 million year ago), and unconsolidated alluvial and colluvial sediments of Quaternary origin (Hall et al. 2008). A total of 90% of the Quaternary sediments are comprised of sedimentary rock around the study site, and a wood sample buried in the sediments showed a radiocarbon age of 34,300 years (Jacobson 1970).

Two 1-ha plots in an evergreen rain forest were established in the tropical lower montane zone between 1200 and 2000–2350 m asl (Kitayama 1992). One was located on Quaternary sediments at approximately 1860 m (hereafter young-soil plot). As explained earlier, the age of the sediments are approximately 35 thousand years old, meaning that the soil in the young-soil plot was younger than that age. The other was located on the Tertiary sedimentary rock at approximately 1600 m (hereafter old-soil plot). Assuming that soil formation initiated after the uplift of Mount Kinabalu, which probably commenced approximately eight million years ago (Hall et al. 2008), the age of the soil in the old-soil plot would be the order of millions of years old. Thus, by comparing the two plots, one can examine the effects of pedogenesis from young (< 34,300 years old) to old (millions of years old) soils formed from essentially the same parent material.

Both plots (200 m × 50 m) were established on gentle (generally < 30°), terrace-like slopes distant from the permanent rivers, such that the longer sides were parallel to contour lines. The topographic map of the old-soil plot was shown by Aiba et al. (2004). The two plots are only 2 km apart in horizontal distance. The altitudinal difference between two plots (260 m) corresponded to a 1.4 °C difference in annual mean temperature (Kitayama et al. 1999).

The soil in the young-soil plot had a less clear differentiation between A and B horizons with angular gravels at various depths, and was classified as Inceptisols. The soil in the old-soil plot appeared to be at a later stage of pedogenesis, with an indication of podozolization, and was classified as Inceptisols/Spodosols. Soil aging resulted in decreased soil pH and translocation of clay as well as Fe and Al (hydro) oxides in the old soil (Fujii et al. 2012). Notably, the old-soil plot had less total and available phosphorus than the young-soil plot (Table 1). Kitayama et al. (2004) demonstrated that soil nitrogen mineralization in the old-soil plot was limited by phosphorus deficiency, leading to lower productivity. The old-soil plot exhibited greater use efficiency of both phosphorus and nitrogen, but lower light use efficiency than the young-soil plot at the stand level.

**Table 1:** Mean ± SD for the soil characteristics ≤ 15 cm depth and use efficiency of nutrients and light in the two 1 ha study plots in a tropical montane rain forests on young versus old soils on Mount Kinabalu after Kitayama et al. (2004), except where otherwise noted

Soil age	Altitude (m)	Slope (°)	pH (H_2_0)^a^	Organic carbon (%)^b^	Total nitrogen (%)^b^	Nitrogen mineralization (μg g^−1^ 10 day^−1^)^c^	Soluble phosphorus (g m^−2^)^d^	Labile organic phosphorus (g m^−2^)^e^	Nitrogen use efficiency (g g^−1^)^f^	Phosphorus use efficiency (g g^−1^)^f^	Light use efficiency (mg mol^−1^)^g^
Young	1860	15	4.2 ± 0.24	7.2 ± 1.0	0.56 ± 0.09	7.79 ± 1.11	0.40 ± 0.37	13.6	110	4272	179
Old	1600	17	4.0 ± 0.09	4.4 ± 1.2	0.32 ± 0.06	1.05 ± 2.28	0.08 ± 0.01	3.5	136	6644	166

^a^Measured using a 1:1 fresh soil to deionized water solution

^b^Based on oven-dried weight

^c^Determined by the buried bag method (Takyu et al. 2002)

^d^Extracted with NH_4_F–HCl solution

^e^≤ 30 cm depth, extracted with NaHCO_3_ solution (Kitayama 2013)

^f^Ratio of annual litterfall mass to the annual content of respective nutrients (Vitousek 1982)

^g^Ratio of above-ground net primary productivity to the annual photosynthetically active radiation absorbed by forest stand

The climate of Mount Kinabalu is typically wet except during occasional droughts associated with El Niño events (Aiba and Kitayama 2002). Mean annual rainfall and mean annual temperature at 1560 m were 18.3 °C and 2380 mm respectively during the period 1996–1997, which included the 1997–1998 El Niño drought (Kitayama et al. 1999*).*

### Field methods and analysis

In both plots, all stems of trees and lianas ≥ 15 cm girth at breast height or 1.3 m above the ground have been measured for girth biennially since 1995 (Aiba and Kitayama 1999). Diameter at breast height (dbh) was calculated as girth divided by π. Multiple stems were tallied separately. Trees with buttresses were measured above the protrusion; the point of measurement was moved upward as buttresses grew for some stems, which were excluded from the growth analysis. Trees were identified to species, and indeterminate species were distinguished as ‘morphospecies.’ No attempt was made to identify liana species. The species list for the 1997 census was given by Aiba et al. (2002).

In this paper, the data for stems ≥ 5 cm dbh for 10 years (2007–2017) were used in order to remove the long-term effects of the 1997–1998 El Niño drought (Aiba and Kitayama 2002; Aiba et al. 2010). Lianas were excluded from analysis except where otherwise noted. Tree height was measured for subsamples of stems in 1996 and 1998 in the old- and young-soil plots, respectively. The description of forest composition and structure was based on the 2007 data except where otherwise noted. To evaluate the dominance, the relative basal area (RBA, %) was calculated as the proportion of the taxa in the sum of the cross-sectional area (π/4 × dbh^2^) of all trees in the plot.

To characterize light conditions, photosynthetically active radiation (PAR) was measured at 20 points systematically arranged in each plot in 2017. Two sets of a pocket-sized PAR logger (DEFI2-L, JFE Advantec Co., Nishinomiya) attached to a 15-m telescopic rod were prepared. One set was placed in a low-canopy site near the plots such that the sensor was exposed above the canopy. The other set was used for measurements in the plots at 1, 3, 5, 7, 9, 11, 13 and 15 m above the ground. We used branches of nearby trees for support to keep the rod upright and the sensor level. At each height, PAR was recorded every second over 60 s under an overcast sky. The mean value of relative light intensity (%, PAR in understory divided by PAR above canopy multiplied by 100) was calculated for each height using the 20 points. The data for cumulative plant surface area from the canopy top, measured by a ground-based LIDAR (light detection and ranging) system, were cited from Aiba et al. (2013).

To evaluate light conditions of individual tree stems in the local canopy structure, the crown position index (CPI, Aiba and Kohyama 1997; Aiba et al. 2004) was assessed for each stem in 2017. The CPI had four grades: upper-canopy stems whose crowns were almost fully exposed vertically (score 1); lower-canopy stems whose crowns were at least partly exposed vertically (score 2); upper-understory stems whose crowns reached the lower foliage of the canopy but were entirely overtopped by the surrounding canopy stems (score 3); and lower-understory stems whose crowns were well below the canopy (score 4). Small stems could have an index 1 or 2 if they were located in canopy gaps. Based on CPI, light environments of stems were dichotomized into exposed (index 1 and 2) versus shaded (index 3 and 4) conditions. We used the ratio of stems with exposed crowns as an index of shade tolerance of species (a higher ratio indicated lower tolerance). The effects of lateral light obliquely coming from canopy openings were not considered, in order to distinguish only those stems with distinctly improved light conditions under exposed conditions.

The following diversity indices were calculated: Fisher’s *α*, Shannon–Wiener index, the reciprocal of Simpson’s index and dominance index (Condit et al. 1996). The Shannon–Wiener index was calculated for both the number of stems (N) and basal area (BA). The dominance index was the largest of the relative abundance (stem number) of species. Percentage similarity, defined as the sum of smaller proportions of abundance for species shared by two plots (Whittaker 1952), was calculated for both the number of stems and BA. Of the 25 most abundant families in the floristic analysis of lowland forests of Borneo (Slik et al. 2003), ten families (Anacardiaceae, Annonaceae, Burseraceae, Dipterocarpaceae, Ebenaceae, Leguminosae, Meliaceae, Myristicaceae, Sapindaceae and Sapotaceae) were considered as characterizing the lowland forest. The four families Fagaceae, Guttiferae (Clusiaceae and Calophyllaceae), Lauraceae and Myrtaceae, as well as Magnoliaceae, Pentaphylacaceae and Podocarpaceae, which were not among the above 25 families, were considered as characterizing montane (including lower-montane) forests (Aiba and Kitayama 1999; Aiba et al. 2015; Slik et al. 2009). No such characterization was attempted for other families.

A Kormogorov–Smirnov two-sample test was used to compare dbh distributions of the two plots; an approximate test based on the frequency distribution was used because of the presence of many ties (Sokal and Rohlf 1995). A two-sample *t* test was used to examine the difference in mean PAR at given height between plots by using Welch’s approximation when the assumption of equal variances was violated. A Chi-square test was used to test whether the frequencies of stems with exposed crowns versus shaded crowns differed between plots in the two dbh classes (5–10 and ≥ 10 cm). For abundant species (see below), a Chi-square test was also used to test whether the frequencies of stems with exposed crowns versus shaded crowns differed from those expected from all species combined.

Aboveground biomass was estimated using the pantropical equation of Chave et al. (2014), incorporating wood density and tree height. Wood density, defined as oven-dry mass divided by wet volume, was measured for dominant species using wood samples collected by an increment borer (Tatsuyuki Seino, Naoki Okada, Yuki Tsujii, unpublished data). The number of samples per species varied from one to seven, and the length of the sampling also varied. For other species, the data (average if two or more values were reported) were extracted from the database of Chave et al. (2009); average genus values were used for species that were not found in the database. For genera not included in the database, plot-specific average values were used. Wood density weighted by BA was calculated for each plot to characterize the biomass investment of wood at the stand level (Lawton 1984). Tree height (*H*, m) was estimated from dbh (*D*, cm) by using the expanded allometric equation (Aiba and Kohyama 1997):

$$H = { 1}/\left( {AD^{h} } \right) \, + { 1}/H^{*} ,$$

where *A*, *h* and H* were plot-specific parameters. H* corresponds to asymptotic tree height. The three parameters of the equation were determined by nonlinear regressions using dbh in 1995 and tree height in 1996 for the old-soil plot and dbh in 1997 and tree height in 1998 for the young-soil plot, after both dbh and height were ln-transformed. Outliers with exceptionally low height (broken stems and heavily inclined stems) were excluded from the analysis.

Growth rate (cm year^−1^) of each stem was calculated as absolute difference in dbh between 2007 and 2017 divided by 10. At the stand level, growth rates increased with increasing dbh and showed strongly positively skewed distributions (i.e. normality assumption violated); thus two dbh classes (5–10 and ≥ 10 cm) were distinguished and a non-parametric test (Wilcoxon two-sample test) was used to examine the difference between two plots in different crown conditions (exposed versus shaded). For individual species, the shape of growth trajectories across tree size can be convex (hump-shaped), concave, or linear (Hara et al. 1991; Hérault et al. 2011). Thus, for eight abundant species (N ≥ 20 in 2007) in both plots, quadratic equations (representing nonlinear patterns) were fitted to the relationship between dbh and growth rates, and then linear equation (representing linear patterns) were fitted when the second-order term was not significant at *P* > 0.05. In the latter case, the significance of linear regression was checked to see if the slope was positive or not significantly different from zero (no species had a negative slope). If linear regressions were significant for both plots, an analysis of covariance (ANCOVA with dbh as a covariate) was used to assess the difference between plots. For species analysis, mean growth rates were calculated for dbh classes with increasing width with increasing dbh (class boundaries: 5, 6, 8, 11, 15, 20, 26, 33, 41, 50, 60, 71 and 83 cm dbh) to improve the heteroscedasticity of data (positive skewness at given dbh and larger variance at larger dbh). Classes that included only one stem were combined with a smaller class (or with a larger class if the 5–6 cm class had one stem only). Mean dbh of stems included in a given class was used to represent dbh of that class. Crown conditions were not distinguished in the species analysis because of small sample sizes.

We also examined if maximum dbh decreased in the old-soil plot compared to that in the young-soil plot for the eight abundant species. The maximum tree size tended to increase with sample size. A tree census was conducted in a larger area (3.24 ha) on sedimentary rock including the old-soil plot (Aiba et al. 2006). Therefore, it was considered that the smaller maximum dbh on the old soil with the larger sample area represented the true difference rather than sampling effects.

## Results

### Forest composition and structure

The old-soil plot had a greater number of tree taxa (48 families, 78 genera and 135 species, including one species of tree fern) than the young-soil plot (41 families, 64 genera and 105 species, all of which were seed plants). Diversity indices also indicated greater diversity on the old soil than on the young soil (Table 2). Notably, the young-soil plot was strongly dominated by *Cinnamomum grandis* (Lauraceae) in terms of the number of stems. Myrtaceae and Fagaceae were the top two dominant families in terms of RBA in both plots (Table 3). Lauraceae and Podocarpaceae were the third dominant families (in RBA) in the young- and old-soil plot, respectively, with greatly reduced dominance in the opposite plot. Podocarpaceae was represented by only three stems (two species) in the young-soil plot.

**Table 2:** Diversity characteristics of trees ≥ 5 cm dbh in the two 1 ha study plots on young versus old soils

Soil age	Number of stems	Number of taxa			Fisher’s *α*	Shannon–Wiener		Reciprocal Simpson	Dominance^a^
		Family	Genus	Species		N	BA		
Young	1806	41	64	105	24.3	3.68	3.67	20.6	0.16
Old	1708	48	78	135	34.4	4.19	3.94	41.0	0.06

^a^Relative abundance of the most abundant species in terms of number of stems

**Table 3:** Relative basal area (RBA, %) and principal habitat of the ten most dominant families in each of the two study plots on young versus old soils

Family	Principal habitat	Young	Old
Myrtaceae	Montane	21.2	25.4
Fagaceae	Montane	17.3	17.4
Lauraceae	Montane	14.1	2.7
Pentaphylacaceae	Montane	7.4	1.8
Magnoliaceae	Montane	6.9	1.4
Sapotaceae	Lowland	6.8	9.3
Clusiaceae	Montane	3.7	6.2
Myristicaceae	Lowland	3.6	1.2
Picrodendraceae	–	1.9	–
Theaceae	–	1.8	1.1
Podocarpaceae	Montane	1.1	13.7
Elaeocarpaceae	–	0.9	2.3
Meliaceae	Lowland	–	5.7
Celastraceae	–	–	1.6
Other families		13.2	10.2

‘–’ Indicates the habitat characterization was not attempted or the absence of the family. Families are arranged in descending order by RBA in the young-soil plot and in descending order by RBA in the old-soil plot for species absent in the young-soil plot

Most of the families characterizing tropical lowland forests either did not occur (Annonaceae, Burseraceae, Dipterocarpaceae, Leguminosae and Meliaceae) or exhibited lower dominance (Anacardiaceae and Sapotaceae) on the young soil. Exceptions were Ebenaceae and Sapindaceae, which occurred only on the young soil, and Myristicaceae, which exhibited much higher dominance on the young soil. Among families that characterize montane forests, Lauraceae, Pentaphylacaceae and Magnoliaceae exhibited decreased dominance on the old soil, whereas Podocarpaceae and Clusiaceae exhibited increased dominance on the old soil.

The two plots shared 67 (39%) of 173 species in total, which corresponded to 64% and 50% of the young- and old-soil plot, respectively. Percentage similarities in terms of the number of stems and the BA between plots were 36% and 32%, respectively. Of these shared species, 32 and 35 species had higher and lower dominance (RBA), respectively, on the old soil, respectively. The change in species dominance in RBA largely mirrored the pattern in family dominance (Table 4). For example, species of Lauraceae, Pentaphylacaceae and Magnoliaceae had lower dominance, whereas species of Myrtaceae (genus *Tristaniopsis*), Podocarpaceae and Clusiaceae had higher dominance, on the old soil. Notably, there were no *Tristaniopsis* species on the young soil. Species of some taxa (Sapotaceae, *Syzygium* in Myrtaceae and *Lithocarpus* in Fagaceae) exhibited variable responses across plots (lower, higher or similar dominance on the old soil).

**Table 4:** Relative basal area (RBA, %) of the ten most dominant species in each of the two study plots on young versus old soils

Species	Family	Young	Old
*Madhuca endertii* H. J. Lam	Sapotaceae	**6.8**	1.3
*Ternstroemia magnifica* Stapf ex Ridl	Pentaphylacaceae	**6.1**	0.4
*Lithocarpus confertus* Soepadmo	Fagaceae	**5.7**	–
*Cinnamomum grandis* Kosterm	Lauraceae	5.5	1.4
*Syzygium steenisii* Merr. & L. M. Perry	Myrtaceae	5.5	0.9
*Lithocarpus lampadarius* (Gamble) A. Camus	Fagaceae	5.3	2.3
*Syzygium* cf. *steenisii* Merr. & L. M. Perry	Myrtaceae	5.2	5.0
*Magnolia carsonii* Dandy ex Noot	Magnoliaceae	4.7	0.2
*Syzygium napiforme* (Koord. & Valeton) Merr. & L. M. Perry	Myrtaceae	3.8	3.8
*Litsea kinabaluensis* Kosterm	Lauraceae	3.6	0.3
*Dacrycarpus imbricatus* (Bl.) de Laub	Podocarpaceae	0.0	3.7
*Garcinia lateriflora* Bl	Clusiaceae	0.0	3.4
*Dacrydium gracilis* de Laub	Podocarpaceae	–	**6.7**
*Tristaniopsis whiteana* (Griff.) P. G. Wilson & J. T. Waterh	Myrtaceae	–	**6.4**
*Payena microphylla* Burck	Sapotaceae	–	**6.4**
*Lithocarpus clementianus* (King) A. Camus	Fagaceae	–	6.0
*Aglaia squamulose* King	Meliaceae	–	3.3
*Tristaniopsis* sp.	Myrtaceae	–	3.1
Other species		47.9	45.5

‘–’ indicates the absence of the species. Species are arranged in descending order by RBA in the young-soil plot and in descending order by RBA in the old-soil plot for species absent in the young-soil plot. The RBAs of the top three dominant species in each plot are in bold

**Fig. 1:**
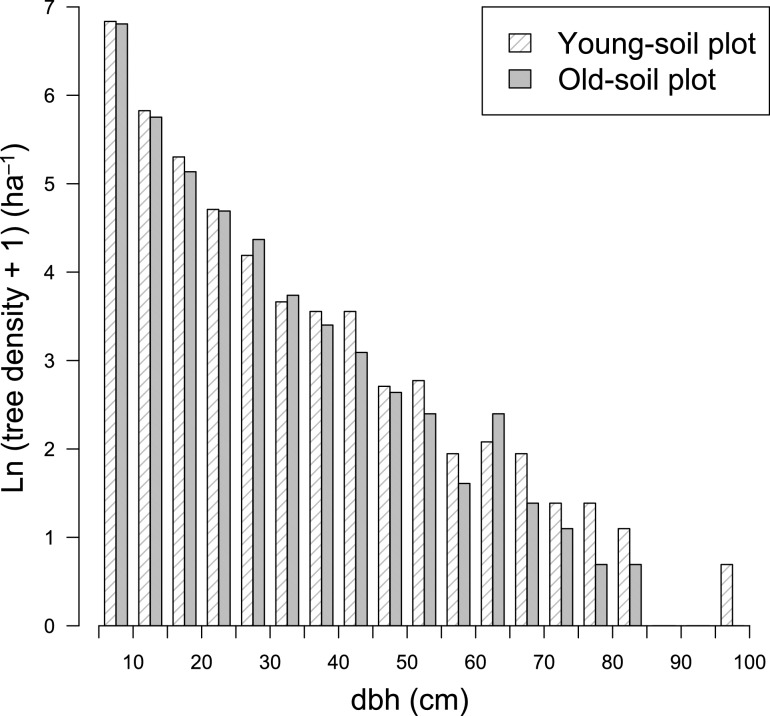
Frequency distributions of dbh of trees in two plots in a tropical montane rain forest over young versus old soils on Mount Kinabalu, Borneo

**Fig. 2:**
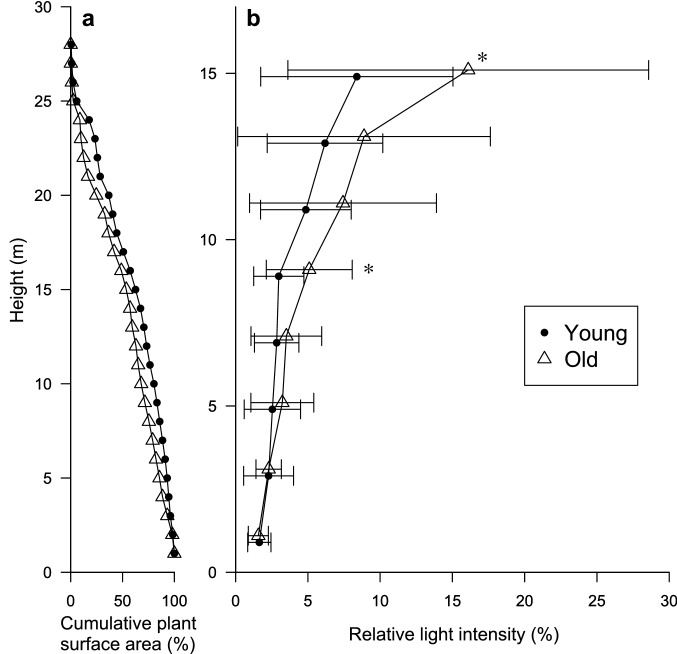
Cumulative plant surface (leaves, branches and trunks) area from the canopy top (**a**) and relative light intensity along the forest profile (**b**) in the young- versus old-soil plots. Error bars indicate standard deviation. *Significant difference (two-sample *t*-test, *P* < 0.05)

The two plots showed no significant difference in dbh distributions (Fig. 1, *P* > 0.05). However, the old-soil plot had fewer stems in all dbh classes (except at 25–35 and 60–65 cm dbh) than the young-soil plot; consequently, the old-soil plot had lower stem density and smaller stand BA than the young-soil plot (Table 5). Both asymptotic tree height (H*) and observed maximum tree height also decreased on the old soil. The average wood density weighted by BA was greater on the old soil than on the young soil (0.59 g cm^−3^ in the young-soil plot versus 0.64 g cm^−3^ in the old-soil plot). The combined effect was a slight decline in aboveground biomass on the old soil: 38.2 kg m^−2^ on the young soil versus 37.0 kg m^−3^ on the old soil. In terms of physiognomy, the old-soil plot had lower densities of buttressed stems and lianas (both 5–10 and ≥ 10 cm dbh) than the young-soil plot did. Relative light intensity was similar at the forest floor (1–3 m above ground), but became increasingly different with increasing height; the old-soil plot was significantly brighter than the young-soil plot at heights of 9 and 15 m above ground (Fig. 2, *P* < 0.05). This reflected a sparser upper canopy in the old-soil plot than in the young-soil plot.

**Table 5:** Structural characteristics of the two 1 ha study plots on young versus old soils

Soil age	Tree basal area (cm^2^ m^−2^)	Mean wood density^a^ (g cm^−3^)	Regression parameters^b^			Observed maximum tree height (m)	Tree aboveground biomass (kg m^−2^)	Density of trees with buttress > 1.3 m height (ha^−1^)	Liana stem density (ha^−1^)		Liana basal area (cm^2^ m^−2^)
			*A* (m cm^−h^)	*h*	H* (m)				5–10 cm	≥ 10 cm	
Young	48.1	0.59	2.24	0.853	37.7	32.1	38.2	48	51	4	0.23
Old	40.9	0.64	2.30	0.916	35.9	30.0	37.0	19	32	2	0.13

^a^Mean wood density weighted by basal area of species (trees only)

^b^Regression parameters for the expanded allometric equation between dbh and tree height

### Growth rate in relation to crown conditions at the stand level

**Fig. 3:**
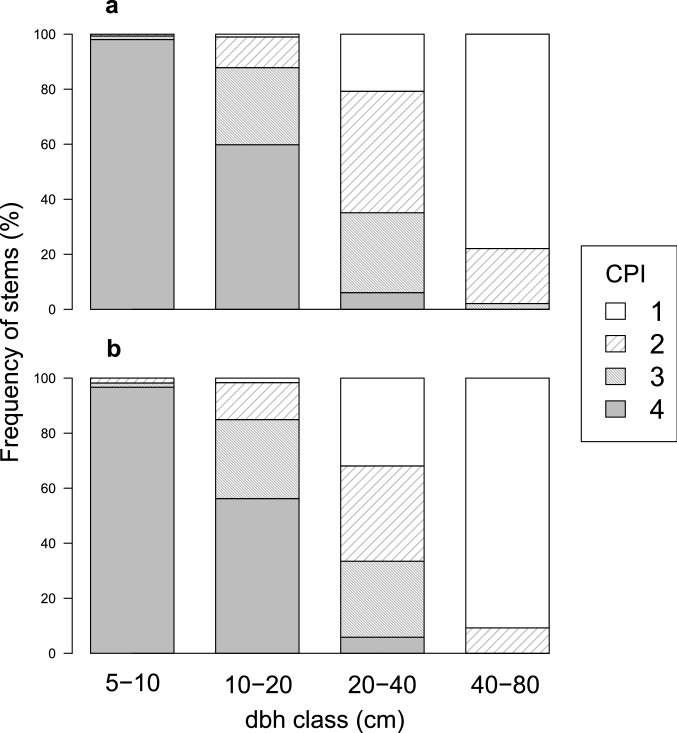
Frequency of tree stems with different index values for the crown position index (CPI) for different dbh classes in the young-soil plot (**a**) and the old-soil plot (**b**). See text for the explanation of CPI

**Fig. 4:**
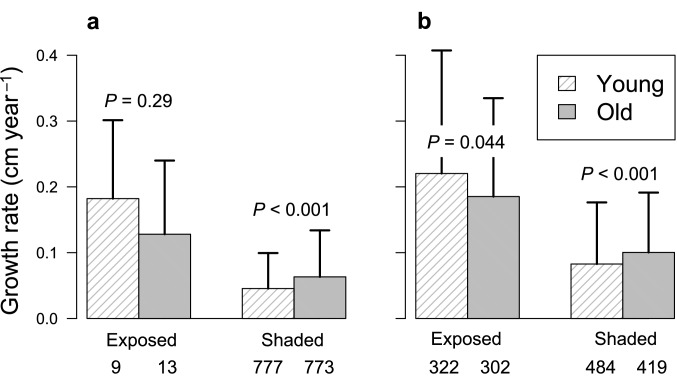
Comparison of the mean absolute growth rate of dbh during 2007–2017 between the young- and old-soil plots for tree stems with exposed crowns (CPI = 1 and 2) and those with shaded crowns (CPI = 3 and 4) at 5 − 10 cm dbh (**a**) and ≥ 10 cm dbh (**b**). Error bars indicate standard deviation. *P* values from Wilcoxon two-sample tests are shown. Numbers below the bars indicate sample sizes

Mean absolute growth rate for all trees ≥ 5 cm dbh was only slightly (6%) greater on the old soil than on the young soil (*P* < 0.001, mean ± SD = 0.093 ± 0.13 cm year^−1^ versus 0.099 ± 0.11 cm year^−1^ on the young versus old soil). The proportions of trees with exposed crowns increased with increasing dbh in both plots (Fig. 3). The frequencies of stems with dichotomized crown positions (exposed versus shaded) did not differ significantly between plots in both the 5–10 cm and ≥ 10 cm dbh classes (*P* > 0.05). In both plots, mean absolute growth rates for dbh increased from 5–10 cm to ≥ 10 cm dbh, and were greater for trees under exposed conditions than for those under shaded conditions (Fig. 4). For trees in exposed conditions, growth rates were greater on the young soil than on the old soil, and the difference was statistically significant for the ≥ 10 cm dbh class (*P* = 0.04), which indicated that trees on the young soil grew faster if they were not limited by light availability. In contrast, for trees in shaded conditions, growth rates were greater on the old soil than on the young soil, and the difference was statistically significant for both dbh classes (both *P* < 0.001), showing that the growth suppression by shading was more severe on the young soil.

### Growth rate, crown position and maximum dbh at the species level

**Fig. 5:**
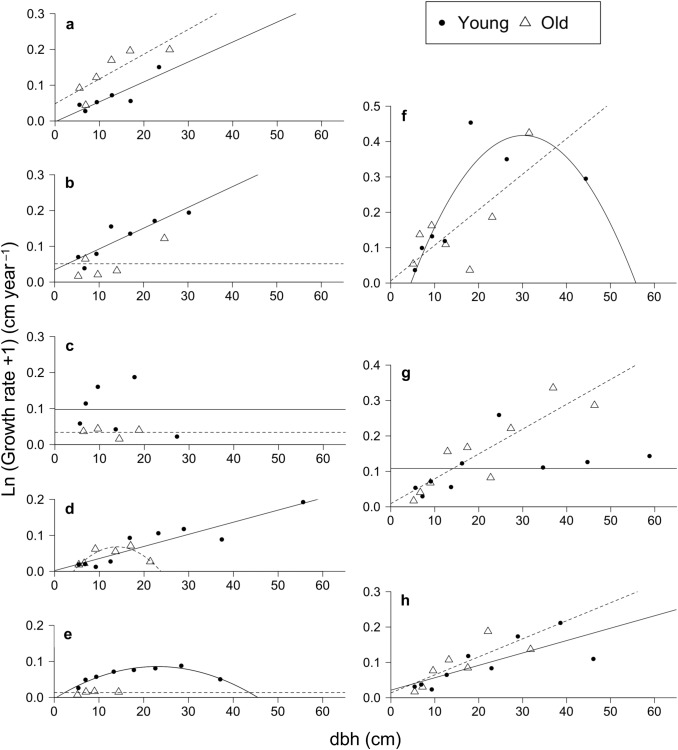
Relationships between initial dbh in 2007 and mean growth rate of dbh (2007–2017) for eight abundant tree species (sample sizes ≥ 20) in the young- and old-soil plots. The eight species were divided into two groups. Group A consists of *Cinnamomum grandis* (**a**), *Garcinia* aff. *eugenifolia* (**b**), *Knema kinabaluensis* (**c**), *Madhuca endertii* (**d**) and *Ternstroemia magnifica* (**e**). Group B consists of *Garcinia* cf. *parvifolia* (**f**), *Syzygium* cf. *steenisii* (**g**) and *Syzygium napiforme* (**h**). See Table 6 for the definitions of the groups. Fitted lines and curves are from linear regression (or mean growth rates when regressions were not significant at *P* < 0.05) and quadratic regression, respectively. Solid lines and curves are for the young-soil plot and dashed ones are for the old-soil plot

The relationships between dbh and growth rate of eight abundant species (Table 6) quantified by quadratic or linear regressions varied across species and plots (Fig. 5). The relationships were linear (including cases where neither regression was significant) or convex; no species exhibited a concave pattern.

**Table 6:** Population characteristics of tree species with sample sizes ≥ 20 in the young- versus old-soil plots

Species	Number of stems		RBA (%)		Maximum dbh (cm)			Ratio of stems with exposed crowns^a^		dbh-growth rate relationships^b^	
	Young	Old	Young	Old	Young	Old	Old^c^	Young	Old	Young	Old
Group A											
*Cinnamomum grandis* Kosterm	294	76	5.5	1.4	27.9	29.1	28.4	0.06***	0.09*	Linear	Linear
*Garcinia* aff. *eugenifolia* Wall	49	29	2.0	1.1	40.9	32.8	34.8	0.21	0.26^†^	Linear	NS
*Knema kinabaluensis* J. Sinclair	43	20	1.1	0.5	28.0	21.6	21.5	0.05**	0.14^†^	NS	NS
*Madhuca endertii* H. J. Lam	130	76	6.8	1.3	66.1	21.5	35.5	0.20	0.06**	Linear	Convex
*Ternstroemia magnifica* Stapf ex Ridl	108	31	6.1	0.4	44.6	20.0	22.0	0.24	0.04*	Convex	NS
Group B											
*Garcinia* cf. *parvifolia* (Miq.) Miq	23	21	1.7	1.3	46.3	34.9	31.0	0.32^†^	0.33^†^	Convex	Linear
*Syzygium* cf. *steenisii* Merr. & L. M. Perry	38	40	5.2	5.0	79.4	55.7	66.6	0.47***	0.36*	NS	Linear
*Syzygium napiforme* (Koord. & Valeton)	79	78	3.8	3.8	49.8	42.3	41.4	0.26	0.23	Linear	Linear
Merr. & L. M. Perry											

Group A includes species that exhibited reduced relative basal area (RBA) by approximately 50% or more in the old-soil plot compared to that in the young-soil plot, whereas group B includes species that exhibited similar RBAs in the two plots

^a^Results of Chi-square tests comparing expected and observed frequencies for all species combined; *P < 0.05; **P < 0.01; ***P < 0.001; ^†^sample size too small for the test

^b^Convex, quadratic relationship with convex curve; linear, linear relationship with positive slope; NS, neither linear nor quadratic regression was significant

^c^Maximum dbh observed during 1997–1999 in a total area of 3.24 ha in the similar environments (the same soil type and similar elevations, Aiba et al. 2006)

Among five species that exhibited greatly reduced dominance (almost 50% or more reduction in RBA) on the old soil compared to on the young soil (species group A), four species (*Garcinia* aff. *eugenifolia*, *Knema kinabaluensis, Madhuca endertii* and *Ternstroemia magnifica*) exhibited lower growth rates on the old soil at least at the larger dbh values. *Cinnamomum grandis* consistently exhibited increased growth rates on the old soil (ANCOVA; slopes, *P* = 0.64; intercepts, *P* = 0.004).

Among three species that had similar dominance (both in number of stems and RBA) in the two plots (species group B), *Syzygium napiforme* exhibited similar mean growth rates at all dbh values (ANCOVA; slopes, *P* = 0.46; intercepts, *P* = 0.40). *Syzygium* cf. *steenisii* showed similar growth rates at < 33 cm dbh values but higher growth rates were observed on the old soil at larger dbh values. *Garcinia* cf. *parvifolia* showed highly variable growth rates at > 15 cm dbh and the difference between plots was unclear. In summary, species of group B exhibited divergent dbh-growth rate relationships, and did not exhibit a growth decline on the old soil.

The ratio of stems with exposed crowns changed little across the two plots for each species (Table 6). Four out of the five species of group A had significantly smaller ratios than expected from all species combined (0.21 in both plots) in at least one of the two plots, ranging from 0.05 to 0.26. The three species of group B tended to have larger ratios than expected for all species combined (with a significant difference for *S*. cf. *steenisii* in both plots), ranging from 0.23 to 0.47. These results suggested that species in group A were more shade-tolerant than those in group B.

Maximum dbh was smaller on the old soil than on the young soil except for *C. grandis*. This was the case even when the maximum dbh in the larger area (3.24 ha) of the old-soil forest was examined. This contrast was especially manifested for two species of group A (*M. endertii* and *T. magnifica*; maximum dbhs were more than doubled on the young soil), for which sampling efforts were similar between soil types, judging from sample areas and stem density in the 1 ha plots.

## Discussion

The two study plots, differing greatly in soil age, were both dominated by Myrtaceae and Fagaceae. The dominance of Podocarpaceae increased on the old soil. Podocarpaceae are associated with infertile soils in both tropical and temperate rain forests (Turner and Cernusak 2011). Podocarpaceae characterized the later stage of a long-term soil chronosequence of temperate rain forests otherwise dominated by angiosperm trees in New Zealand, which is analogous to the situation in the present study (Coomes et al. 2005, 2009; Mason et al. 2012). In contrast, Lauraceae decreased in dominance with soil aging in the present study. This is consistent with a study conducted in a 7.5-ha plot of tropical montane forest in northern Thailand, which showed that the majority of Lauraceae species were associated with lower slopes where soils are fertile (Sri-Ngernyuang et al. 2003). The absence or scarcity of many lowland families (Anacardiaceae, Annonaceae, Burseraceae, Dipterocarpaceae, Leguminosae, Meliaceae and Sapotaceae) in our young-soil plot could be explained by higher elevation (i.e. colder climate). However, some lowland families (Ebenaceae, Myristicaceae and Sapindaceae) had higher dominance or occurred only in the young-soil plot, which could have occurred because their distributions were limited by reduced nutrient availability (as an indirect effect of low temperature) rather than by direct effects of colder climate.

The often-cited diagram of tropical vegetation zones in the Malay Peninsula (Whitmore 1984) depicts Fagaceae and Lauraceae dominating the tropical lower montane forest which may be called the ‘lower montane oak-laurel forest’ (Ashton 2003, 2014). The young-soil plot on Mount Kinabalu with high dominance of Lauraceae may deserve such a name. However, a literature review showed that the dominance of Fagaceae and Lauraceae appeared to be restricted to mountains with nutrient-rich soils affected by volcanism (e.g. the Philippines, Sumatra and Java), and Myrtaceae and conifers were more dominant on non-volcanic mountains with nutrient-poor soils typically found on Borneo and the Malay Peninsula (Aiba 2016). Lower montane forests on infertile soils characterized by the dominance of Myrtaceae (*Tristaniopsis* in particular) and conifers, such as one in our old-soil plot, may be called ‘lower montane kerangas’ (Ashton 2014) because of the structural and floristic resemblance to tropical heath forests (kerangas) on lowland podosols (Brunig 1974; Miyamoto et al. 2015).

In the present results, the young-soil plot had a higher density of lianas and more frequent occurrence of buttressed trees, which characterize the physiognomy of tropical lowland rain forest (Grubb 1977). Thus, in accordance with some of the floristic characteristics, the young-soil plot showed physiognomy that was more similar to the lowland forests than that shown by the old-soil plot, despite the young-soil plot being located at a higher altitude. The BA was much greater in the young-soil plot than in the old-soil plot (48.1 versus 40.9 cm^2^ m^−2^), yet aboveground biomass was only slightly greater in the young-soil plot (38.2 versus 37.0 kg m^−2^). Trees on the young soil had buttresses > 1.3 m high far more often than trees on the old soil. As the diameter of buttressed trees was measured at > 1.3 m height, their calculated dbh underestimated the real dbh, causing an underestimation of aboveground biomass. Therefore, the decline of aboveground biomass with soil aging should in reality be greater than was indicated by the present results.

The decline of tree aboveground biomass, along with decreased liana abundance, with soil aging should lead to decreased leaf area in the upper canopy on the old soil, which is consistent with the pattern in the optically measured leaf area index (Kitayama et al. 2004). Indeed, the old-soil plot was brighter than the young-soil plot at the understory > c. 5 m height. The dominance of the conifer *Dacrydium gracilis* with needle leaves may also have contributed to the sparse upper canopy in our old-soil plot (Ushio et al. 2017). This could explain why the understory trees grew faster on the old soil under shaded conditions (Fig. 4), which concurred with the pattern found in a chronosequence of a New Zealand rain forest (Coomes et al. 2009). However, light conditions at the forest floor at 1–3 m height were similar between plots (Fig. 2), in accordance with our earlier measurements using hemispherical photographs (Sawada et al. 2016). The field observations suggest that the abundant rattans (climbing palms) with diameter < 5 cm in the old-soil plot may account for this.

Among eight species abundant in both plots, seven species had smaller maximum sizes on the old soil, suggesting that soil nutrient availability limited tree size. Two groups of species (A and B) showed different growth responses to soil aging (Fig. 5, Table 6). Four of the five species (except *C. grandis*) of group A (which exhibited reduced dominance on the old soil) had lower growth rates on the old soil at least when they grew larger. This suggests that they are nutrient-demanding species that suffer from nutrient deficiency as the whole-plant nutrient demand increases with plant size. In contrast, three species of group B (which exhibited similar dominance in the two plots) seemed to tolerate nutrient deficiencies because they did not exhibit a growth decline on the old soil. However they were less tolerant of shade than species in group A, which was suggested by relatively high ratios of stems with exposed crowns. More shade-intolerant species such as conifers and *Tristaniopsis* were virtually absent in the understory on the young soil. Thus, along the soil-age gradient, the dominant species would change from shade-tolerant, nutrient-demanding species (such as group A) on the young soil, via relatively shade-intolerant species (such as group B), to shade-intolerant species that are highly tolerant of nutrient deficiencies (such as conifers and *Tristaniopsis*) on the old soil. It should be noted that this pattern of species turnover is for the dominant species only. Light-demanding species (such as conifers and *Tristaniopsis*) do occur at low abundance on the young soil (by regenerating in rare events of gap formation), which results in a wider spectrum of shade tolerance on more fertile soils (Coomes et al. 2009; Dent and Burslem 2016).

Chronosequence studies may be confounded by site-specific factors unless environmental factors other than age are held constant across sites. In this study, the 260-m elevational difference between plots may have been problematic. The limited distribution of Quaternary sediments and the precipitous topography of Kinabalu Park prevented us from exactly controlling the elevations of the two plots. The young-soil plot was located at a higher elevation than the old-soil plot. Faster growth of canopy trees (Fig. 4), better developed aboveground structure (Table 5) and greater productivity (Kitayama et al. 2004) on the young soil despite a colder climate could, therefore, be reasonably attributed to soil differences. The lesser dominance of Podocarpaceae on the young soil is also counter to the general trend in tropical forests where Podocarpaceae increases in dominance at higher elevations (Slik et al. 2009; Turner and Cernusak 2011).

Based on the general characteristics of habitat association at family and genus levels, it was considered that soil was the most important factor causing floristic differences between the two plots. Analysis of tree growth indicated that the growth of understory trees was limited by light on the young soil, whereas that of canopy trees was limited by nutrients on the old soil. The eight species abundant in both plots showed differential responses to soil aging. In summary, the present results generally supported the scenario of floristic turnover with soil aging presented in the introduction. Therefore, we suggest that the differential responses of species to decreasing nutrient availability with a concomitant increase in understory light levels explain floristic turnover with soil aging in this species-rich forest.
